# Use of personalized graft cage and RIA bone graft in a case of acute infection following a grade 2 open tibial shaft fracture: a new option for bone grafting in patients with critical size segmental bone defects

**DOI:** 10.1093/jscr/rjaf493

**Published:** 2025-07-16

**Authors:** Christian Thomas Hübner, Anna Veenstra, Felix Karl-Ludwig Klingebiel, Tobias Bayer, Vincent Landre, Sandro-Michael Heining, Christian Hierholzer, Hans-Christoph Pape

**Affiliations:** Department of Trauma, University Hospital Zurich, University of Zurich, Raemistrasse 100, 8091 Zurich, Switzerland; Department of Trauma, University Hospital Zurich, University of Zurich, Raemistrasse 100, 8091 Zurich, Switzerland; Department of Trauma, University Hospital Zurich, University of Zurich, Raemistrasse 100, 8091 Zurich, Switzerland; Department of Trauma, University Hospital Zurich, University of Zurich, Raemistrasse 100, 8091 Zurich, Switzerland; Department of Trauma, University Hospital Zurich, University of Zurich, Raemistrasse 100, 8091 Zurich, Switzerland; Department of Trauma, University Hospital Zurich, University of Zurich, Raemistrasse 100, 8091 Zurich, Switzerland; Department of Trauma, University Hospital Zurich, University of Zurich, Raemistrasse 100, 8091 Zurich, Switzerland; Department of Trauma, University Hospital Zurich, University of Zurich, Raemistrasse 100, 8091 Zurich, Switzerland

**Keywords:** tibial fractures, bone defects, graft cage, infections, intramedullary fracture fixation, Masquelet technique, trauma

## Abstract

Critical size segmental bone defects represent a challenging problem. Recently, personalized, structured three-dimensional graft cages have been developed to improve ingrowth of autologous bone material and reduce resorption. A 44-year-old patient presented with an open tibial shaft fracture after a motorcycle incident with minor bone loss. Following intramedullary nailing, an acute infection developed that required removal of ~4 cm bone in a circular mode. After multiple interventions, a personalized 3D-printed graft cage was implanted and filled with autologous bone material, harvested by intramedullary reaming from the ipsilateral femur. Ten months postoperatively, the patient is completely pain free and shows good clinical and radiological status. In conclusion, use of a 3D-printed graft cage in combination with Reamer Irrigator Aspirator (RIA) might represent a new additional feature for larger bone defects to avoid gravity or perfusion related resorption of large area defects.

## Introduction

Fracture healing in patients with critical size segmental bone defects continues to represent a challenging problem, especially in open fractures or associated with infections [1]. In the past, many different techniques such as distraction by circular frames or unilateral external fixation devices, or intramedullary lengthening devices have been advocated [2].

The Masquelet technique provides certain improvements, as the induced membrane facilitates the ingrowth of any bone graft (i.e. heterologous, autologous, or combined materials) [3]. Following the development of generation of bone harvesting via the medullary canal, the technique has frequently been combined with Reamer Irrigator Aspirator (RIA) reaming [4].

However, in larger long bone defects, bone resorption has been described [5], which concurs our own experience [6]. As a result, it has been thought that the resorption in larger defects may be a result of gravity induced secondary motion of the graft. Moreover, it was thought that although the RIA provides adequate volumes to cover larger size defects, a combination with a structured graft material might be of use [7]. A graft cage has been developed as a result of these issues and represents an individualized 3D-printed implant that is combined with small particle bone graft. The implant is composed of 96% polycaprolactone, a bioresorbable polymer, and 4% hydroxyapatite with a degradation rate of 2–4 years. A calcium phosphate coating is intended to promote mineralization and osteointegration [8].

So far, no larger series have been described. This manuscript summarizes a complex case where application of autologous bone harvested by RIA II is filled with a 3D printed polycaprolactone cage in a case of an infected open tibial fracture.

## Case presentation

A 44-year-old patient was admitted to a local hospital after a motorcycle-accident, where an open displaced tibial shaft was diagnosed. Acute intramedullary nailing was performed using a coated implant, as the fracture was graded as II° open. The soft tissues were closed primarily.

Approximately one month later the patient presented to the emergency room and reported oozing soft tissues. Acute wound infection was diagnosed. The work-up revealed a deep infection involving the implant (*S. aureus* and *Enterobacter cloacae*).

Implant removal and temporary external fixation was performed, the wound was washed out several times and closed again. Five weeks later the soft tissues showed no healing—instead, a persistent infection was diagnosed, thus leading to removal of the external fixator and placement of the fracture in a splint.

One week later, an extended wound debridement was performed, the medullary canal was reamed, and a new coated tibial nail (Expert PROtect, size 12 × 375 mm, DePuy Synthes) was implanted. The soft tissue defect was treated with a vacuum-system (VAC).

**Figure 1:**
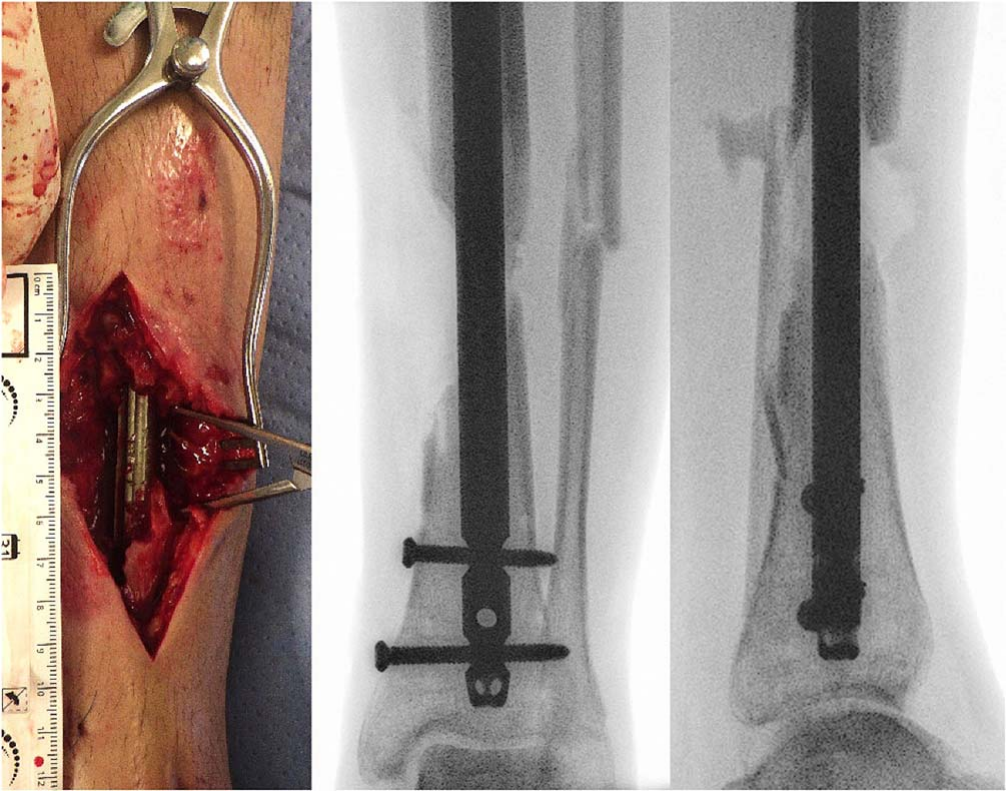
(a) Photography of intraoperative bone defect before graft cage implantation and (b) intraoperative X-rays of left lower extremity show segmental bone defect.

The patient then referred himself to our unit and we performed further extensive debridement of soft tissues and necrotic bone in repeated steps (Fig. 1). During the second revision, placement of gentamycin–polymethylmethacrylate antibiotic cement spacer was performed.

The workup showed an osseous defect of 4 cm laterally and 6 cm medially. The soft tissues required treatment as a 9 x 3cm measuring defect zone appeared. As all three arterial vessels (peroneal and anterior and posterior tibial arteries) were intact, an anterolateral thigh flap (ALT-flap) was applied to cover the soft tissue defect. The patient was discharged to a rehabilitation unit.

Three months after cement spacer implantation and an interval of five days sparing antibiotics, microbiologic sampling was done, which was negative.

**Figure 2:**
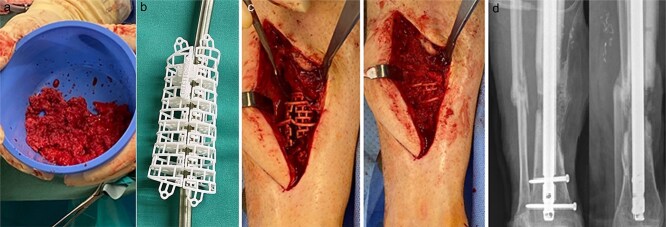
(a) RIA-harvested bone graft; (b) graft cage; (c) operation site with graft cage; (d) postoperative X-ray after graft cage implantation.

Autologous bone from the ipsilateral femur was harvested using the RIA II-system (11 mm and 12.5 mm reamer head) (Fig. 2a). The predesigned graft cage (TRUMATCH™ Graft Cage – Long Bone, DePuy Synthes, Zuchwil, Switzerland), was inserted and filled with autologous bone (Fig. 2b and c). Postoperatively, the patient was allowed touch down partial weight bearing.

The follow-up at 8 weeks showed a good clinical and radiographic outcome. About 6 weeks later the patient presented with acute pain due to failure of a proximal locking screw, which was replaced. About 50% weight bearing was initiated at 6 months post operative, followed by full weight bearing ~6 weeks thereafter. About 8 months after the implantation of the graft cage the patient presented without walking aids and the gait analysis showed a symmetrical gait.

**Figure 3:**
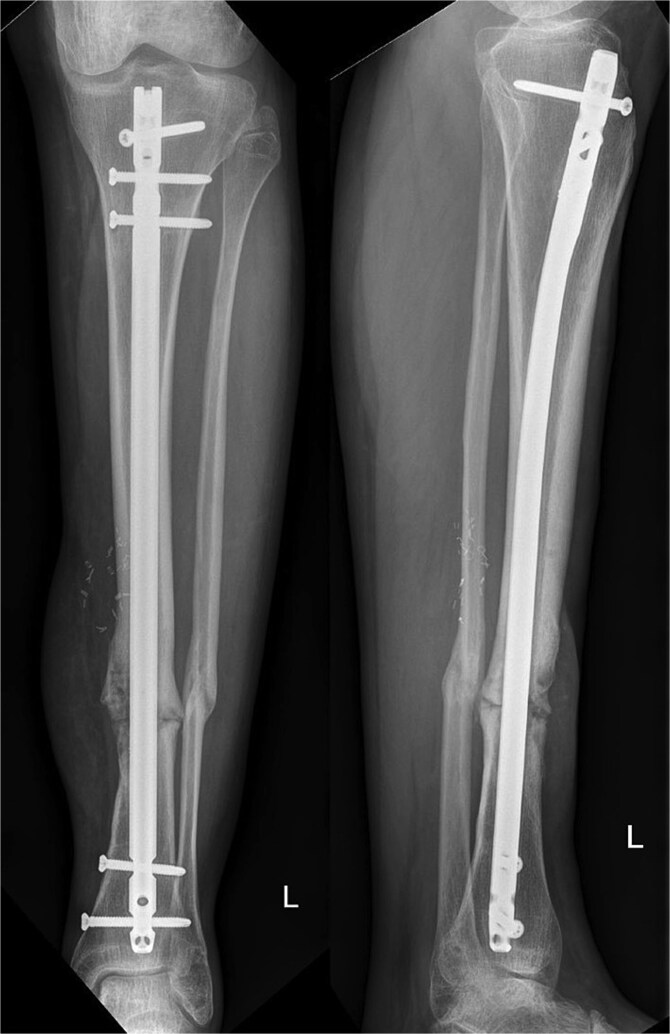
X-ray of lower extremity after 10 months.

Ten months postoperatively, the patient was pain-free and demonstrated a normal gait pattern, associated with radiological signs of osseous consolidation (Fig. 3).

**Table 1:** Time course of treatment

**Time line**	**Clinical treatment and decisions**	**Soft tissue status**	**Bony status**
Day 1	Tibia fracture, acute debridement, ***IM nailing wound closure***	Second degree open	2 cm defect
Day 12	Discharge to rehabilitation unit	Closed	
4 weeks	Readmission for purulent infection ***Removal IM Nail, conversion ex fix***	Open	
2 months	***Removal ex fix and splint application*** *VAC treatment*	Open	
2 m 7 days	Debridement, ***Implantation Expert Tibia PROtect Nail,** VAC treatment medial wound*	VAC	
2 m 14 days	Relocation hospital *Radical debridement*	VAC	Triangular defect with 4 cm lateral and 6 cm medial
2 m 17 days	Debridement, ***implantation gentamycin cement space,** VAC treatment*	VAC	Triangular defect with 4 cm lateral and 6 cm medial
2 m 21 days	*Debridement, VAC-Exchange*	VAC	
2 m 26 days	*VAC-Removement*, ***ALT-Flap***	Closed with ALT-Flap	
4 m 1 week	Clinical control	non-irritant wound conditions, vital ALT-Flap	
5 m 2 weeks	***Exchange prox. Locking-screws*** Microbiologic sampling, Infection with *Staphylococcus caprae*	Closed	6 cm defect, gentamycin-spacer
6 m 1 week	***Exchange cement spacer*** Microbiologic sampling, negative results	Closed	Gentamycin spacer
6 m 3 weeks	***RIA right femur, removal cement spacer, implantation Trumatch graft***	Closed	4.5 cm defect, TRUMATCH graft cage
7 m	Demission to rehabilitation care	Closed	
7 m 5 days	Removement sutures	Closed	
7 m 2 weeks	***Operative removement splinters femur***		
8 m	Ambulant consultation, 15 kg weight bearing	Closed	Beginning consolidation
8 m 3 weeks	Ambulant consultation, 40 kg weight bearing	Closed	
9 m 6 days	Ambulant consultation, full weight bearing	Closed	
9 m 9 days	Ambulant consultation, broken proximal locking screw	Closed	
9 m 2 weeks	***Exchange proximal locking screw***	Closed	
10 months	Ambulant consultation 40 kg weight bearing	PICO7-Treatment	
10 m 1 week	Ambulant consultation 40 kg weight bearing	PICO7-Treatment	
10 m 4 weeks	Ambulant consultation, 40 kg weight bearing	Closed	Progredient consolidation
12 months	Ambulant consultation, full weight bearing	Closed	Progredient consolidation
14 months	Ambulant consultation, full weight bearing, painfree	Closed	Progredient consolidation, especially medial

Operations are written in italics. Relevant procedural steps are marked in bold.

## Discussion

Critical size segmental defects in long bone represent a challenging problem and have a sustained socioeconomic burden [2]. Multiple operations and prolonged hospital stays are often required before satisfactory results can be achieved. Distraction osteogenesis may represent an effective solution, but implies a certain risk of secondary complications. Among them, nonunion of the docking site has been named [9]. Another disadvantage, both in in ring fixators and unilateral external fixation, are pin-site infections, which have been described in substantial (between 9% and 100%) numbers [10].

To address the issues of repeated interventions, single stage options imply distraction nails, which exist as mechanic or magnetic options. They appear to be associated with better patient compliance and lower psychosocial stress. However, the high implant costs and inability to use an intramedullary device in the face of infections are of concern [11].

Hydroxyapatite, other artificial substances and the incorporation of the cement has been examined [12]. Unfortunately, the ingrowth of any of these substances has been proven insufficient. Especially in large autografts, migration has been described. The Masquelet technique might represent a solution in the face of closed tissue and no further surgery.

The clinical background behind Masquelet’s assumptions towards implications of vascularity and issues of the Masquelet membrane has been explained in detail by his coworker Pelissier. In particular, the proof of active secretions of vascular components appears to be promising [13].

As far as large grafts are concerned, the issue of resorption has long been described [14].

In fact, the AO RIA task force, which was installed to develop the second generation of the RIA reamer, has been provided the task of clinically evaluation the options for a graft that is now in clinical use [15].

Our patient presented with an excellent result after 10 months without any restrictions and was completely pain-free. Our radiographic analysis demonstrated that no resorption occurred and no secondary movement of the graft (Fig. 3). Moreover, the even distribution of the graft was associated with no heterotopic bone formation or issues that would be related to donor site pathologies in distraction osteogenesis.

The combination of RIA and 3D-printed graft cages may offer several advantages, like supporting the harvested bone to structurally ingrowth in the region of its placement, minimize soft tissue complications, as only a single step procedure is required compared to distraction device methods.

## Conclusion

In conclusion, 3D-printed graft cages might represent a new additional feature for patients with larger bone defects. Our first experience showed stable graft positioning, followed by uneventful ingrowth and excellent clinical results. These aspects will have to be proven in future clinical studies involving a larger number of patients.
